# Successful uterine-sparing surgical management in a patient with a large multi-fibroid uterus

**DOI:** 10.1093/jscr/rjab233

**Published:** 2021-06-26

**Authors:** Lucy M Holden

**Affiliations:** Department of Obstetrics and Gynaecology, Joondalup Health Campus, Joondalup, Australia

## Abstract

Uterine fibroids are a common gynaecological condition. A key factor when deciding on surgical approach for fibroid management is a patient’s age and desire for fertility with uterine-sparing treatment generally recommended in women who are aiming for future pregnancies. This case report discusses a woman who presented with a very large multi-fibroid uterus. In this case, a hysterectomy was recommended due to safety concerns however, this advice was declined as the patient desired uterine preservation. Fertility testing showed that the patient was not peri-menopausal and abdominal myomectomy was safely performed to good effect. This case report outlines the surgical approach undertaken, in addition to considerations for fertility and pregnancy management.

## INTRODUCTION

Uterine fibroids, or leiomyomas, are benign smooth muscle tumours arising from cells of the uterine myometrium. They are a common gynaecological condition, affecting up to 80% of pre-menopausal women [1]. Although a large number of women with fibroids are asymptomatic, those with symptoms often report significant negative impact on their quality of life [2]. Symptomatic women will often seek specialist opinion to discuss surgical treatment of their fibroids. Gold standard treatment for uterine fibroids is hysterectomy, or myomectomy in women who desire uterine preservation, however less invasive techniques have recently been developed. In women of child-bearing age who wish to have their fertility preserved, treatment planning can be complex in patients presenting with multiple large fibroids, particularly if hysterectomy would be the safer approach. This case report describes a 43-year-old female with a large multi-fibroid uterus causing mass effect symptoms, who desired uterine preservation.

## CASE REPORT

A 43-year-old Australian female presented to the emergency department complaining of shortness of breath, urinary frequency and heavy menstrual bleeding on a background of known multi-fibroid uterus. She denied any bowel symptoms. The patient had been diagnosed with a large multi-fibroid uterus whilst living in the USA, however, due to a lack of health insurance and high medical costs, surgical treatment had not been sought. She had received two blood transfusions in 2018 due to anaemia. Due to the coronavirus disease of 2019 pandemic she decided to return home to Australia and upon her return presented to the emergency department seeking treatment. Her body mass index was 36 and she had iron deficiency anaemia secondary to heavy menstrual bleeding. She had no past surgical history. She had never been pregnant and desired preservation of her fertility. She was on oral iron supplementation.

**Figure 1:**
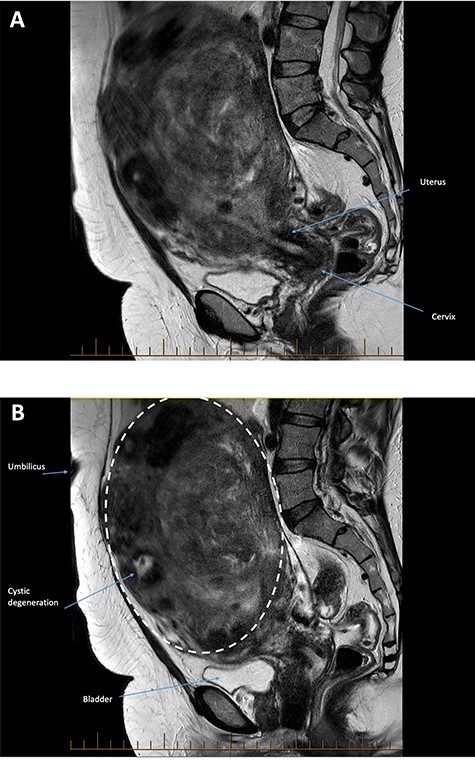
(**A** and **B**): Sagittal images from a T2 weighted sequence. Marked enlargement of the uterus secondary to fibroids. The largest fibroid arises at the uterine fundus (dotted line) and extends into the upper abdomen above the level of the umbilicus. It contains areas of focal high T2 signal reflecting cystic degeneration. No malignant or aggressive features are present.

On examination, her vital signs were within normal limits. Her uterus was palpable to just below the xiphisternum and her abdomen was mildly tender. A full blood count was performed which showed haemoglobin of 91 g/l with a mean corpuscular volume of 73 fL. Renal function was normal. A pelvic ultrasound revealed a uterus that was grossly enlarged by multiple fibroids, extending above the level of the umbilicus and into the right hypochondrium. The largest fibroid was a broad-based right fundal exophytic subserosal fibroid measuring 147 × 114 × 180 mm. There was a large posterior intramural fibroid measuring 124 × 111 × 103 mm, in addition to multiple other fibroids. The patient was further assessed with magnetic resonance imaging (MRI; Fig. 1A and B), which re-demonstrated marked uterine enlargement secondary to the fibroids, some of which demonstrated areas T2 hyperintensity reflecting cystic degeneration. The largest exophytic fibroid at the right lateral uterine fundus (dotted outline) extended superiorly to abut the inferior right hepatic lobe, compressing the right kidney and displacing bowel loops to the left upper quadrant. The largest intrauterine fibroid compressed the endometrial cavity and displaced it rightward. No malignant or aggressive features were present. These imaging findings were consistent with an ultrasound and MRI performed in the USA ~18 months prior.

The patient was commenced on tranexamic acid and Provera to manage her bleeding while awaiting surgery. A hysterectomy was advised, however due to her desire for children this recommendation was declined by the patient. To investigate her fertility anti-mullerian hormone, follicle stimulating hormone and luteinizing hormone levels were performed (all within normal parameters). The patient elected to undergo a myomectomy with the understanding the there was a risk of proceeding to hysterectomy intraoperatively. An iron transfusion was organized preoperatively.

An abdominal uterine myomectomy was performed. A midline laparotomy was made and extended above the umbilicus due to the size of the uterus. Three uterine incisions were made (fundal, posterior wall and anterior wall) and a total of 29 fibroids were removed ranging from 20 cm to 1 cm in diameter. The cavity was breached with the posterior incision and closed with 2-0 polydioxanone suture; otherwise, the uterine wounds were closed with multi-layered 1.0 vicryl sutures. The uterus remained large (20 weeks gestation) at the end of the procedure. On further inspection no large fibroids were noted and diffuse adenomyosis was diagnosed. Estimated blood loss from the procedure was 1.5 l.

Haemoglobin was 74 g/l post-operatively and one unit of packed red bloods cells was transfused. The patient had an uneventful recovery and was discharged home on day five post-operatively. Histopathology showed benign leiomyomas with no atypical features. The patient was reviewed in clinic 6 weeks after her surgery. She noted one period post-operatively and described this as normal. She expressed interest in a sperm donor program and was referred to a fertility service for further management.

## DISCUSSION

Desire for future pregnancy will always be a key driver in choice of management for fibroids. Uterine-sparing surgery is generally recommended in women of child-bearing age in an effort to preserve fertility, with minimally invasive surgical techniques preferred over hysterectomy. In those desiring fertility, myomectomy is generally recommended over uterine artery embolization (UAE). This is due to UAE being associated with a higher rate of re-intervention, higher likelihood of intrauterine adhesions [3] and a general deficit in evidence regarding fertility and pregnancy outcomes [4, 5]. In this case, hysterectomy was recommended due to the size of the patient’s fibroids and safety concerns, particularly regarding intraoperative blood loss. As this patient declined hysterectomy due to her desire for fertility, testing to determine the patient’s fertility status was of particular importance. Had her blood results shown a peri-menopausal state, then the desire for uterine preservation may have been moot. Ultimately, the surgical team worked to respect the patient’s wishes for fertility preservation with referral to fertility services at the completion of her treatment.

In regards to future pregnancy, there are multiple considerations when presented with a patient with fibroids. The effect of fibroids themselves on fertility is poorly understood and optimal management is debatable. Subserosal fibroids are unlikely to impair fertility, however submucosal and intramural fibroids are associated with decreased fertility and increased rates of miscarriage [6, 7]. In patients with known fibroids prior to pregnancy or diagnosed antenatally, there are implications for their care. Foetal growth surveillance is recommended due to the risk of intrauterine growth restriction [8]. There is an additional increased risk of preterm birth and caesarean delivery [9]. Careful determination of fibroid location in early pregnancy is also important to ensure that fibroids do not present an obstruction and vaginal delivery is possible. If a caesarean section is required, knowledge of fibroid location allows for careful surgical planning. Should this patient become pregnant, she will need close surveillance and careful delivery planning under specialist care.

## References

[1] Baird DD, Dunson DB, Hill MC, Cousins D, Schectman JM. High cumulative incidence of uterine leiomyoma in black and white women: ultrasound evidence. Am J Obstet Gynecol 2003;188:100e7. 12548202 10.1067/mob.2003.99

[2] Fortin C, Flyckt R, Falcone T. Alternatives to hysterectomy: the burden of fibroids and the quality of life. Best Pract Res Clin Obstet Gynaecol 2018;46:31–42. 29157931 10.1016/j.bpobgyn.2017.10.001

[3] Tanos V, Berry KE. Benign and malignant pathology of the uterus. Best Pract Res Clin Obstet Gynaecol 2018;46:12–30. 29126743 10.1016/j.bpobgyn.2017.10.004

[4] Clements W, Ang WC, Law M, Goh GS. Treatment of symptomatic fibroid disease using uterine fibroid embolisation: an Australian perspective. Aust N Z J Obstet Gynaecol 2020;60:324–9. 31956995 10.1111/ajo.13120

[5] Karlsen K, Hrobjartsson A, Korsholm M, Mogensen O, Humaidan P, Ravn P. Fertility after uterine artery embolisation of fibroids: a systematic review. Arch Gynecol Obstet 2018;297:13–25. 29052017 10.1007/s00404-017-4566-7

[6] Klatsky PC, Tran ND, Caughey AB, Fujimoto VY. Fibroids and reproductive outcomes: a systematic literature review from conception to delivery. Am J Obstet Gynecol 2008;198:357. 18395031 10.1016/j.ajog.2007.12.039

[7] Kroon B, Johnson N, Chapman M, Yazdani A, Hart R. Fibroids in infertility – consensus statement from ACCEPT (Australasian CREI Consensus Expert Panel on Trial Evidence). ANZJOG 2011;51:289–95. 21806566 10.1111/j.1479-828X.2011.01300.x

[8] Radhika BH, Naik K, Shreelatha S, Vana H. Case series: pregnancy outcome in patients with uterine fibroids. J Clin Diagn Res 2015;9:QR01–4. 10.7860/JCDR/2015/14375.6621 PMC4625296 26557577

[9] Karlsen K, Schioler Kesmodel US, Mogensen O, Humaidan P, Ravn P. Relationship between uterine fibroid diagnosis and the risk of adverse obstetrical outcomes: a cohort study. BMJ Open 2020;10:e032104. 10.1136/bmjopen-2019-032104 PMC7044982 32071172

